# Hemiplegia, Following Ligature of the Common Carotid

**Published:** 1844-05-04

**Authors:** 


HEMIPLEGIA, FOLLOWING LIGATURE OF THE COMMON CAROTID.

Dr. Fairfax adds another case to the list of those in which this accident has happened. His patient, a middle-aged lady, much reduced by chronic disease of the lungs, died five days after the operation. The mental faculties continued perfect to the last. The hemiplegia afflicted the right side, not the face, and was not observed until an hour after the operation.—*Ibid.*

